# Histone Acetylation-Mediated Regulation of the Hippo Pathway

**DOI:** 10.1371/journal.pone.0062478

**Published:** 2013-05-06

**Authors:** Dipanjan Basu, Miguel Reyes-Múgica, Abdelhadi Rebbaa

**Affiliations:** Department of Pathology, University of Pittsburgh and the Children’s Hospital of Pittsburgh of UPMC, Pittsburgh, Pennsylvania, United States of America; The University of Texas Health Science Center, United States of America

## Abstract

The Hippo pathway is a signaling cascade recently found to play a key role in tumorigenesis therefore understanding the mechanisms that regulate it should open new opportunities for cancer treatment. Available data indicate that this pathway is controlled by signals from cell-cell junctions however the potential role of nuclear regulation has not yet been described. Here we set out to verify this possibility and define putative mechanism(s) by which it might occur. By using a luciferase reporter of the Hippo pathway, we measured the effects of different nuclear targeting drugs and found that chromatin-modifying agents, and to a lesser extent certain DNA damaging drugs, strongly induced activity of the reporter. This effect was not mediated by upstream core components (i.e. Mst, Lats) of the Hippo pathway, but through enhanced levels of the Hippo transducer TAZ. Investigation of the underlying mechanism led to the finding that cancer cell exposure to histone deacetylase inhibitors induced secretion of growth factors and cytokines, which in turn activate Akt and inhibit the GSK3 beta associated protein degradation complex in drug-affected as well as in their neighboring cells. Consequently, expression of EMT genes, cell migration and resistance to therapy were induced. These processes were suppressed by using pyrvinium, a recently described small molecule activator of the GSK 3 beta associated degradation complex. Overall, these findings shed light on a previously unrecognized phenomenon by which certain anti-cancer agents may paradoxically promote tumor progression by facilitating stabilization of the Hippo transducer TAZ and inducing cancer cell migration and resistance to therapy. Pharmacological targeting of the GSK3 beta associated degradation complex may thus represent a unique approach to treat cancer.

## Introduction

The Hippo pathway is a novel signaling cascade first reported to play a key role in regulation of organ size [1], [2], [3], [4], [5]. It was identified in Drosophila through screening for genes whose loss of function leads to tissue overgrowth, which resulted in identification of *warts,* also called *lats,* as a gene associated with the most pronounced phenotype [6]. Subsequent studies indicated that loss of *Warts/Lats accelerates* cell cycle progression and inhibits apoptosis [7], [8], [9] suggesting that this gene may have a tumor suppressor function. During the last few years, several upstream and downstream mediators of the Hippo pathway have been identified including NF2, RASSF, MOB, MST*1/2*, WW45, 14-3-3, YAP, TAZ and TEAD [1] and the list is still growing [10]. Core components of the Hippo kinase cascade (Mst/Lats) are conserved in mammalian genomes and have been shown to act in tandem with casein kinase 1 epsilon (CK1ε) to induce phosphorylation-mediated inhibition of the Hippo transducers YAP, TAZ and TEAD [11], [12]. It was shown for instance that phosphorylation of Hippo transducers facilitates their binding to14-3-3 and subsequent cytoplasmic sequestration [13], [14], [15]. Other studies have demonstrated that sequential phosphorylation and degradation of TAZ is facilitated by GSK3 beta/CK1ε [16], [17] suggesting that alternative mechanisms of regulation may exist. As a result of these perturbations, several biological processes, including cell-fate determination [18], mitosis [19], and pluripotency [20] could be affected. Of particular interest, deregulation of the Hippo pathway was found to be associated with carcinogenesis [21]. This is best illustrated by studies in which *lats1* knockout in mice led to soft tissue sarcomas and ovarian stromal cell tumors [22]. Moreover, expression of TAZ showed an exceptionally strong association with poor patient survival from non-small lung cancer and thyroid carcinoma [23], [24]. Alterations in this gene and/or its molecular partners YAP and TEAD have also been reported in cancers derived from colon, lung, liver or esophagus [25], [26], [27]. The underlying mechanisms by which expression of Hippo transducers facilitate tumor progression are not fully understood however available data indicate that they may act in conjunction with components of Wnt and/or TGF beta signaling pathways [28], [29], [30] to induce certain cancer stem cell related processes such as epithelial to mesenchymal transition (EMT) and the development of resistance to therapy [31], [32], [33].

Based on the demonstrated role of Hippo signaling in cancer progression, approaches to alter its activity may prove to be effective for therapy, however for this to be achieved, prior understanding of the mechanisms that regulate this pathway is critical. Genes implicated in cell-cell interaction are thought to represent major regulators of the Hippo signaling. In fact, mutations of such genes in *Drosophila,* recapitulate the Hippo phenotype [34], [35] and increased phosphorylation and cytoplasmic sequestration of YAP was observed when cultured mammalian cells reach confluency and begin to establish inter-cellular cell contacts [11]. Conversely, disruption of cell–cell junctions resulted in increased nuclear localization of both YAP and TAZ [30]. Interestingly, other membrane components such as the G-protein coupled receptors (GPCRs), with no major role in cell-cell interaction, have also been shown recently to regulate Hippo signaling [36], [37], highlighting the multifactorial aspect of this regulation. While most of research effort up to now was directed towards defining the role of plasma membrane associated molecules in regulating the Hippo pathway, the possibility of its nuclear regulation has not yet been described. Here we set out to verify this possibility and define putative mechanism(s) by which it might occur. Potential consequences of such regulation on cancer cell migration and resistance to therapy, and the role of targeting key components of the Hippo pathway to suppress these processes are also addressed.

## Materials and Methods

Human melanoma (WM115 and WM266), breast cancer (MCF-7), and colon cancer (SW480) cell lines were purchased from ATCC (Rockville MA). The 293 kidney cells were obtained from Clontech (Mountain View, CA). Dulbecco’s Modified Eagle’s Medium (DMEM), MEM, RPMI, Horse serum and fetal bovine serum (FBS) were obtained from BioWhittaker (Walkersville, MD). The following drugs and reagents were obtained from the companies cited: The 8xGTII luciferase reporter construct [33] from Addgene (Cambridge, MA); Doxorubicin, and antibody to beta-Actin from Sigma-Aldrich (St. Louis, MO); CTGF, IL8, Wnt3a from (R&D Systems, Minneapolis, MN); Antibodies to Zeb1and Twist1 from Santa Cruz (Santa Cruz, CA), Vimentin and N-cadherin from Cell Signaling Technology (Danvers, MA); secondary antibodies conjugated to horseradish peroxidase from Jackson Immunoresearch Lab Inc. (West Grove, PA); Enhanced chemiluminescence reagents (ECL) and Immobilon-P transfer membrane for Western blots from Millipore (Bedford, MA). Reagents for DNA transfection were obtained from Life Technologies (San Diego, CA).

### Cell Culture and Transfections

Melanoma and breast cancer cells were cultured in MEM supplemented with 10% FBS as described by the supplier. Colon cancer cells were maintained in RPMI supplemented with 10% FBS, and the 293 cells were cultured in DMEM supplemented with 10%FBS penicillin/streptavidin and non-essential aminoacids (Life Technologies, San Diego, CA). Transfections were carried out in 6 well plates using a lipofectamine kit (Life Technologies, San Diego, CA) as described by the manufacturer. Briefly, 3 µg of DNA were mixed in 100 µl of transfection solution containing 90 µl of serum free culture medium and 10 µl lipofectamine. After 20 min incubation at room temperature, the mixture was added to the wells and incubated for 5 hours. The medium was then replaced with a new one before the inhibitors were added to the corresponding wells and incubated for an additional 24 hours. Protein extracts were harvested and processed for either Western blot or luciferase assay as described below.

### Western Blot

Proteins were extracted from cells cultivated in monolayers using 100 µl of lysis buffer (50 mM HEPES pH 7.4, 150 mM NaCl, 100 mM NaF, 1 mM MgCl_2_, 1.5 mM EGTA, 10% glycerol, 1% Triton X100, 1 µg/ml leupeptin, 1 mM phenyl-methyl-sulfony l-fluoride). For nuclear and cytoplasmic fractionation [38], cells were re-suspended in 400 µl of buffer A (10 mM HEPES pH 7.9; 10 mM KCl;0.1 mM EDTA; 0.1 mM EGTA; 1 mM DTT; 0.5 mM PMSF). The mixture was incubated for 30 min on ice, then 25 µl of a 10% solution of Nonidet NP-40 was added and the homogenate centrifuged for 30 sec. Equal quantities of protein were separated by electrophoresis on a 12% SDS-PAGE gel and transferred to Immobilon-P membranes. Proteins of interest were identified by reaction with specific primary and secondary antibodies linked to horseradish peroxidase and detected by chemiluminescence.

### Q-PCR

Total RNA was extracted using the RNeasy mini kit (Qiagen, Santa Clarita, CA).The first-strand cDNA was synthesized according to manufacturer’s instructions using ThermoScript RT-PCR system (Life Technologies, San Diego, CA). Gene expression was measured by real-time PCR using the Maxima Syber green Master Mix (Fermentas, Glen Burnie, MA) on ABI 7500 instrument (Applied Biosystems, Carlsbad, CA). Q–PCR primers are reported in Table 1. Gene expression was normalized to that of GAPDH used as internal control.

**Table 1:** Primers used in Q-PCR.

Gene	Forward primer 5′–3′	Reverse primer 5′–3′
Wnt3A	CCTGCACTCCATCCAGCTACA	GACCTCTCTTCCTACCTTTCCCTTA
Wnt4	GATGTGCGGGAGAGAAGCAA	ATTCCACCCGCATGTGTGT
Wnt 7B	CCCGGCAAGTTCTCTTTCTTC	GGCGTAGCTTTTCTGTGTCCAT
Wnt 10A	GGCAACCCGTCAGTCTGTCT	CATTCCCCACCTCCCATCT
EGF	CAGGGAAGATGACCACCACT	TTCCCACCACTTCAGGTCTC
IGF1	CCCCACTCACCAACTCATAG	GGTATTTGGGGCCTTTATGT
IL1α	ATCAGTACCTCACGGCTGCT	TGGGTATCTCAGGCATCTCC
SMA	AGTTACGAGTTGCCTGATGG	GAGGTCCTTCCTGATGTCAA
FGF23	TGGGTTAGGTTTTCTGTGGA	AAGAATTTCCAAGGGGATTG
IL-6	ATGCAATAACCACCCCTGAC	GAGGTGCCCATGCTACATTT
IL-8	TAGCAAAATTGAGGCCAAGG	AGCAGACTAGGGTTGCCAGA
CTGF	TTTGGCCCAGACCCAACTAT	GTGCAGCCAGAAAGCTCAAA
TGF-β2	AACAAGAGCAGAAGGCGAAT	TGCCATCAATACCTGCAAAT
GAPDH	GAGTCAACGGATTTGGTCGT	TTGATTTTGGAGGGATCTCG

### TEAD Activity Assay

The 8×GTIIC-luciferase reporter which contains 8 TEAD binding sites was used to measure activation of the Hippo pathway. To evaluate the specificity of this reaction, we used a DNA construct containing luciferase driven by the CMV promoter as a control. These plasmids were transfected transiently into cells using the lipofectamine kit as follows: 3 µg of DNA were mixed in 100 µl of transfection solution containing 90 µl of serum free culture medium and 10 µl lipofectamine. After 20 min incubation at room temperature, the mixture was added to the wells and incubated for 5 hours. The medium was then replaced with a new one before the inhibitors or conditioned medium (CM) from cells exposed to drugs were added to the corresponding wells. After incubation for an additional 24 hours, the cells were lysed and protein extracts used as a source of luciferase. For each test, the luminescence value of CMV driven luciferase was substracted from the one obtained with 8×GTIIC-luciferase. In control samples, this difference is considered as 100% of activity.

### Collection of Conditioned Medium (CM)

Near confluent cultured WM 115 melanoma cells grown in 25 cm^2^ flasks containing MEM and 10% FBS at 37°C in 95% air/5% CO_2_, were exposed to Belinostat at 10^−6^ M for 24 hours. The cell monolayer was then washed three times and placed in fresh medium for 2 hours to allow elimination of intracellular Belinostat. The cells were then placed in 5 ml of new MEM for an additional 24 hours to allow secretion of soluble factors. The medium was harvested and centrifuged at 1000×g for 10 min to remove residual cells and debris. The supernatant was collected and used as conditioned medium (CM) containing Belinostat-induced secreted factors.

### MTT Assay

Cells were incubated in a 96 well plate with the drugs for 96 h. The fraction of viable cells were quantitatively determined by a colorimetric MTT assay as described previously [39]. MTT (10 µl of 5 mg/ml solution) was added to each well of the titration plate and incubated for 4 h at 37°C. The cells were then solubilized by the addition of 100 µl of 10% SDS/0.01 M HCl and incubated for 15 h at 37°C. The optical density of each well was determined in an ELISA plate reader using an activation wavelength of 570 nm and reference wavelength of 650 nm. The percentage of viable cells was determined by comparison with untreated control cells.

### Statistical Analysis

Graph data is presented as mean ± standard error (SE). All analyses were performed by using a 2-way ANOVA and values in the treated samples were compared to the corresponding controls. *P*<0.05 was considered statistically significant. Statistical calculations were performed with SPSS 16.0 for Windows (SPSS, Chicago, IL, USA).

## Results

### Respective Roles of DNA Damage and Chromatin Modification in Regulation of the Hippo Pathway

**Figure 1:**
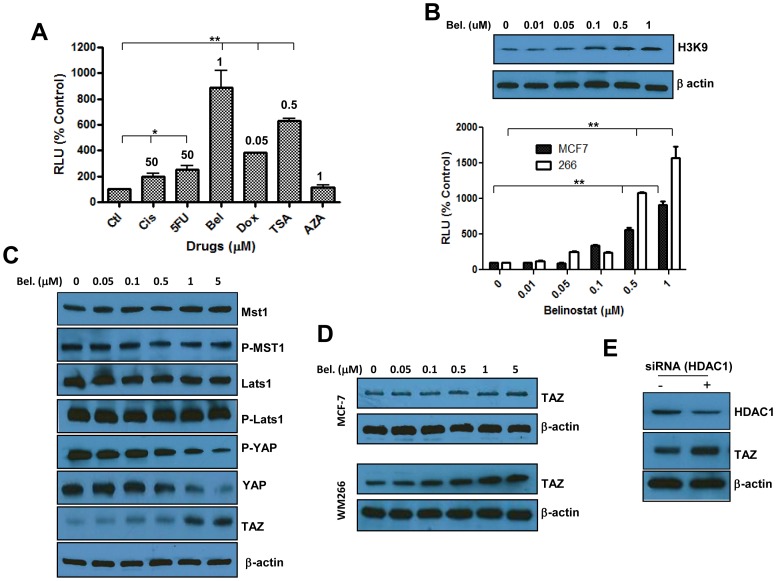
Respective roles of DNA damage and chromatin modification in regulation of the Hippo pathway. Panel A. Hippo reporter activity in response to drugs tested at concentrations that induce 50% inhibition of proliferation in SW480 cells (indicated at the top of each bar). Ctl: control., Cisp: cisplatin., Dox: doxorubicin., Bel: Belinostat., TSA: Trichostatin A., AZA: 5 Azacitidine(decitabine)). Panel B. Western blot showing the effect of Belinostat on acetylation of Histone H3 at Lysine 9 (H3K9) (Upper level), and activity of Hippo reporter in MCF7 and WM 266 melanoma cells. Each bar in Panels A and B represents the average of three determinations ±SE. Statistical significance is shown for drug-treated cells compared to the corresponding untreated controls (*p<0.05, **p<0.001). Panel C. Western blots depicting the effect of Belinostat on expression and/or phosphorylation of various components of the Hippo pathway in SW480 cells. Panel D. Expression of TAZ in MCF7 and WM 266 cells in response to Belinostat. Panel E. Representative data showing the effect of siRNA mediated Knockdown of HDAC1 on expression of TAZ in WM266 cells measured by Western blot.

The effects of DNA and chromatin modulating drugs on activity of the Hippo pathway were analyzed using the (8×GTII) luciferase reporter system [33] in which a DNA binding sequence for TEAD drives expression of the luciferase gene. For this, HEK 293 cells were transfected with this construct and exposed to the DNA damaging drugs doxorubicin, cisplatin and 5FU, the DNA methyltransferase inhibitor 5 AzaC, or histone deacetylase inhibitors TSA and Belinostat, each at a concentration that induce 50% inhibition of cell proliferation. As shown in Figure 1A, the DNA de-methylating agent 5 AzaC has no effect on TEAD reporter activity, however the DNA damaging agents doxorubicin, cisplatin and 5-FU exerted a relatively moderate stimulation (up to 2.5 times increase). In contrast, both histone deacetylase inhibitors Belinostat and TSA induced strong activation of the TEAD reporter. Stimulation of the luciferase activity in response to Belinostat was concentration dependent and correlated with the levels of histone acetylation induced by this drug (Fig. 1B). The effect of Belinostat was also valid in other cell lines (Fig. 1 B) suggesting that this observation may represent a general phenomenon.

To gain insight on potential molecular mediators, we measured expression and phosphorylation levels of various intracellular mediators of Hippo signaling and as shown in Figure 1C, neither expression of Mst1 and Lats1, nor their phosphorylation levels changed significantly. Unexpectedly, phosphorylation of YAP decreased in response to increased concentrations of Belinostat, which could be explained by decreased expression of this gene as noted in Figure 1C. Of particular interest, the levels of the Hippo transducer TAZ increased in a drug concentration-dependent manner in WM115 cells (Fig. 1C), as well as in other cell lines (Fig. 1D). siRNA to HDAC1 resulted in increased levels of TAZ in WM266 cells (Figure 1E) suggesting that this phenomenon is histone acetylation-dependent.

### Regulation of Hippo Downstream Genes by Belinostat and Role of TAZ in Mediating these Effects

**Figure 2:**
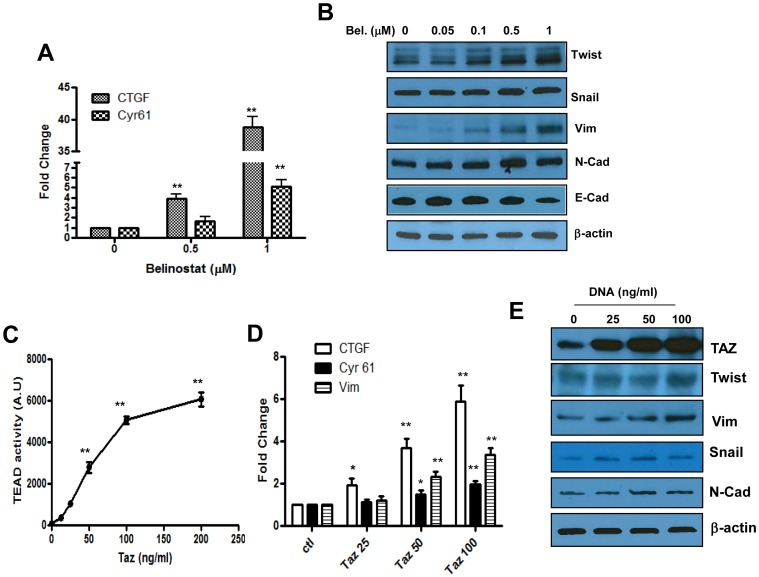
Regulation of Hippo downstream genes by Belinostat and role of TAZ in mediating these effects. Panel A. Expression of TAZ target genes CTGF and Cyr61 measured by Q-PCR in the absence or the presence of Belinostat at the indicated concentrations (µM). Panel B. Representative Western blots showing the expression of EMT genes in response to Belinostat in SW480 cells (Ecad: E Cadherin, N-Cad: N cadherin). Panel C. Effect of TAZ gene overexpression on activity of the Hippo reporter. SW480 cells were transfected with the TAZ DNA construct at the indicated concentrations and activity of luciferase reporter measured after 24 hrs. Panel D. Effect of TAZ overexpression on expression of its downstream target genes. Cells were transfected by TAZ as described in panel C and expression of CTGF and Cyr 61 and Vimentin (Vim) was measured by Q-PCR. Panel E. Representative Western blots showing expression of EMT associated genes in response to TAZ overexpression (Vim: Vimentin, N-Cad: N cadherin). Data in panels A, C and D, represent average of three determinations ±SE. Statistical significance is shown for drug-treated or TAZ-transfected cells compared to the corresponding controls (*p<0.05, **p<0.001).

To better define the relationship between histone acetylation and the Hippo pathway, we measured expression downstream genes in response to Belinostat. The data presented in Figure 2A indicate that expression of CTGF and Cyr61, two well-known targets of TAZ [40], was strongly induced in the treated cells and in a concentration dependent manner. Since the Hippo pathway has been shown to signal for epithelial mesenchymal transition (EMT) through overexpression of TAZ [14], [33], we determined if expression levels of EMT genes are altered in response to Belinostat and if so, whether overexpression of TAZ would be sufficient for inducing such alterations. The results (Fig. 2B) indicate that this was the case since the levels of Twist, snail, Vimentin and N-Cadherin were all induced and this was accompanied by a slight decrease of E cadherin in response to Belinostat. Importantly, TAZ overexpression resulted not only in enhanced TEAD reporter activity (Fig. 2C) and expression of its target genes CTGF, Cyr61 (Fig. 2D) as it might be expected, but also in induction of the same EMT genes induced by Belinostat (Fig. 2E). Together, these findings suggest that cancer cell exposure to histone deacetylase inhibitors may paradoxically signal for cancer progression by facilitating EMT through induction of TAZ and its downstream target genes.

### Mechanism(s) by which Histone Acetylation Regulates the Hippo Pathway

#### a) Induced expression versus stabilization of TAZ

**Figure 3:**
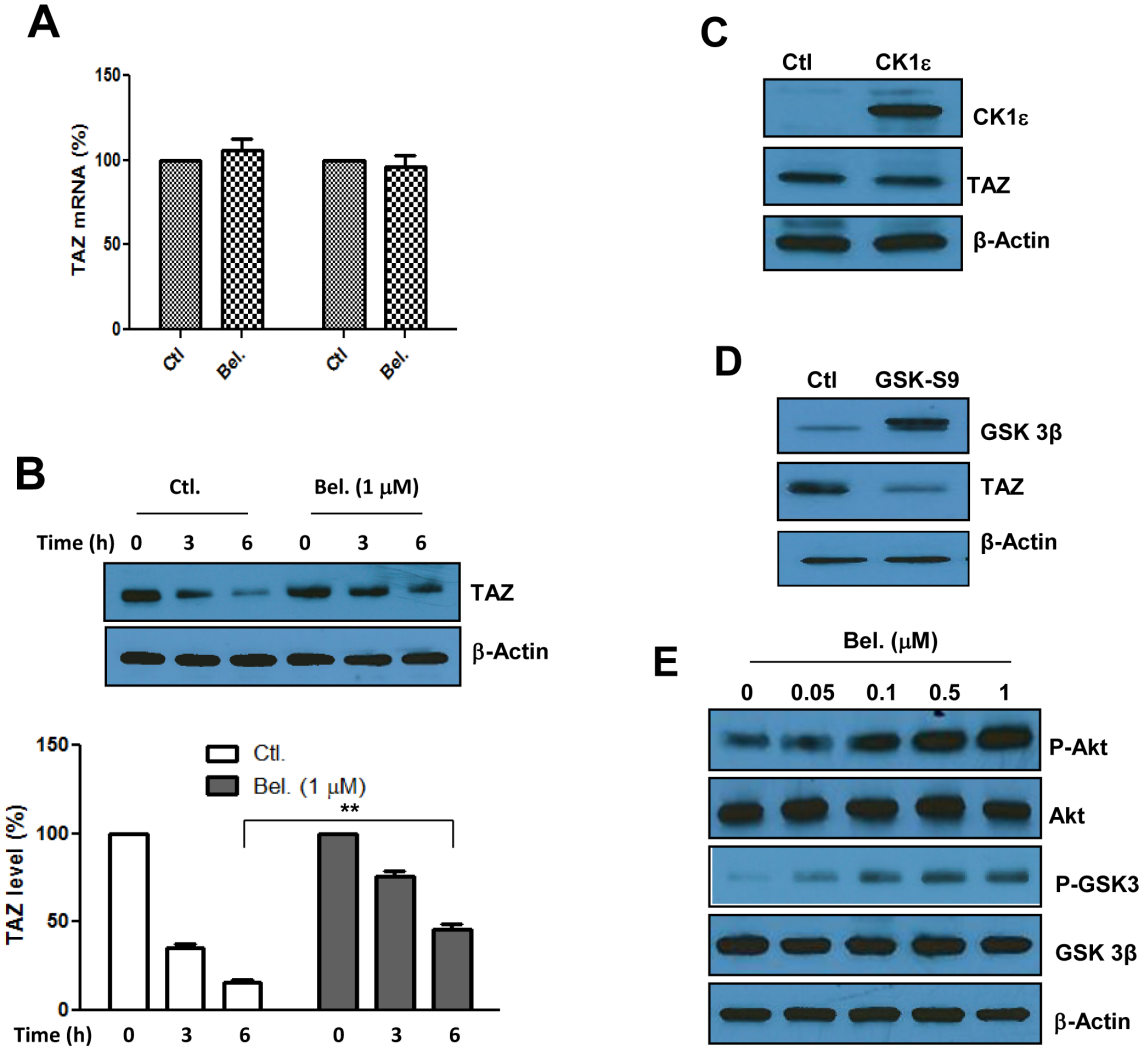
Belinostat-induces stabilization rather than expression of TAZ. Panel A. expression of TAZ in response to Belinostat (5 µM) measured quantitative PCR. Panel B. Effect of Belinostat on TAZ stabilization. SW480 cells were exposed to cycloheximide (CXH) at 10 µM concentration in the absence or the presence of Belinostat (µM). Proteins were extracted at the indicated times after addition of the compounds and TAZ protein levels determined by Western blot. Band densities were quantified by the Image J software (NIH) and graphed. Data in graphs A and B represent average of three determinations ±SE. Significance (p<001) is shown in graph B between Belinostat-treated cells for 6 hours and the corresponding non-treated cells. Panels C and D. SW480 cells were transfected with genes coding for CK1ε or constitutively active GSK3 beta (GSK-S9) and TAZ protein levels determined by Western blot after 48 hours. Panel E. Effect of Belinostat on phosphorylation of Akt and GSK3 beta. The cells were exposed to the drug for 1hour in serum free medium and protein phosphorylation detected by Western blot using specific antibodies. Beta actin is used as a loading control in panels B, C, D and E.

To determine if TAZ regulation by Belinostat occurs at the gene or post-translational level, we first measured its expression using quantitative PCR. No changes were however detected by either technique (Fig. 3A), suggesting that the observed increase in levels of this gene (Fig. 1C) was not due to enhanced RNA expression. To determine if Belinostat inhibits TAZ degradation, protein synthesis was inhibited using cycloheximide and a chase experiment was carried out in the absence or the presence of the drug. As shown in Figure 3B, TAZ was indeed degraded at a slower rate in cells exposed to Belinostat compared to non-treated controls. Since both GSK3 beta [16] and casein kinase 1εhave been shown to play key roles in facilitating TAZ degradation, we sought to determine which one of these two enzymes would be implicated. The results indicate that overexpression of Casein kinase 1ε had only a minimal effect if any on TAZ levels (Fig. 3C), however overexpression of the constitutively active form of GSK3 beta (GSK3-S9) prevented TAZ stabilization (Fig. 3D). In support of this, phosphorylation levels of both GSK 3 beta and its upstream kinase Akt were induced by Belinostat (Fig. 3E). These findings suggest that histone acetylation-mediated induction of TAZ occurs at the post-translational level and may be caused at least in part by inhibition of GSK3 beta associated degradation complex which is known to prime proteins for proteasomal degradation [41].

#### b) Potential role of secreted factors in mediating the effects of histone acetylation on TAZ transactivation

**Figure 4:**
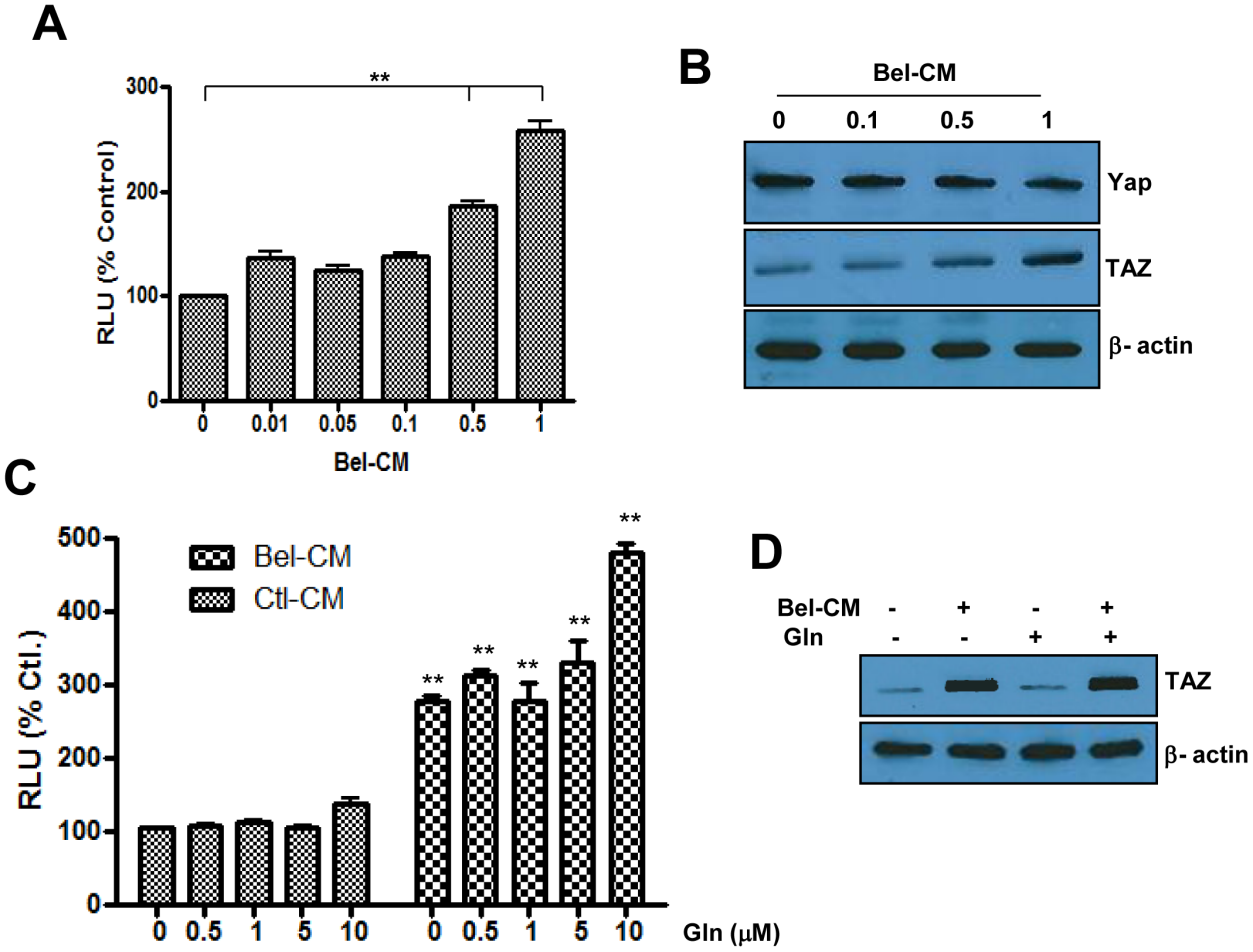
Potential role of G-protein coupled receptors in mediating Belinostat induced activation of the Hippo pathway. Panel A. Effect of conditioned medium from Belinostat pre-exposed cells on activation of the Hippo reporter in naïve cells (not previously exposed to the drug). SW480 cells were incubated with Belinostat at the indicated concentrations for 24 hours, the drug was then washed extensively and the cells incubated with fresh media for 24 hrs. The resulting conditioned media (CM) were then incubated for 24 hrs with naïve cells transfected with the Hippo luciferase reporter, after what the activity of the enzyme was determined. The data represent average of three determinations ±SE. Statistical significance is shown for Bel-CM-exposed cells compared to the control (**p<0.001). Panel B. Representative Western blots showing the effect of CM from Belinostat treated cells on expression of YAP and TAZ in naïve cells. Beta actin was used as a loading control. Panel C. Effect of glucagon (Gln), a GPCR antagonist, on Bel-CM induced activation of the Hippo reporter. SW480 cells transfected with the luciferase reporter were incubated in the absence or the presence of CM from cells pre-exposed to 0.5 µM Belinostat (Bel-CM), and in the absence or the presence of glucagon at the indicated concentrations. Luciferase activity was measured after 24 hrs of incubation. The data represent average of three determinations ±SE. For every Gln concentration, values were compared between Bel-CM exposed cells and those exposed to control CM (**p<0.001). Panel D. Effect of Glucagon on Belinostat-mediated increase in TAZ levels determined by western blot in cells exposed or not to Bel-CM and in the absence or presence of Gln at 5 µM. Staining with beta actin represents a loading control.

To obtain further insights on the mechanisms by which histone acetylation signals for the stabilization of TAZ, we analyzed the potential role of secreted soluble factors which in an autocrine or paracrine manner, could signal for inhibition of GSK3 beta associated degradation complex, resulting in enhanced TAZ accumulation. For this, we first determined if conditioned medium from cells pre-exposed to Belinostat (Bel-CM) induces TEAD reporter activity in naïve cells (not previously exposed to the drug), and the results indicate that this was indeed the case (Fig. 4A). Stimulation levels obtained with Bel-CM (1.2 to 2.5 times) are however lower than those obtained in cells directly incubated with the drug (5 to 10 times, Fig. 1A and 1B), suggesting that a continuous expression and secretion of these factors may be required for higher and sustained reporter activity. Interestingly, Bel-CM also inhibited YAP expression and enhanced TAZ levels (Fig. 4B) in a manner that recapitulates the effects of Belinostat on these two genes (Fig. 1B).

**Figure 5:**
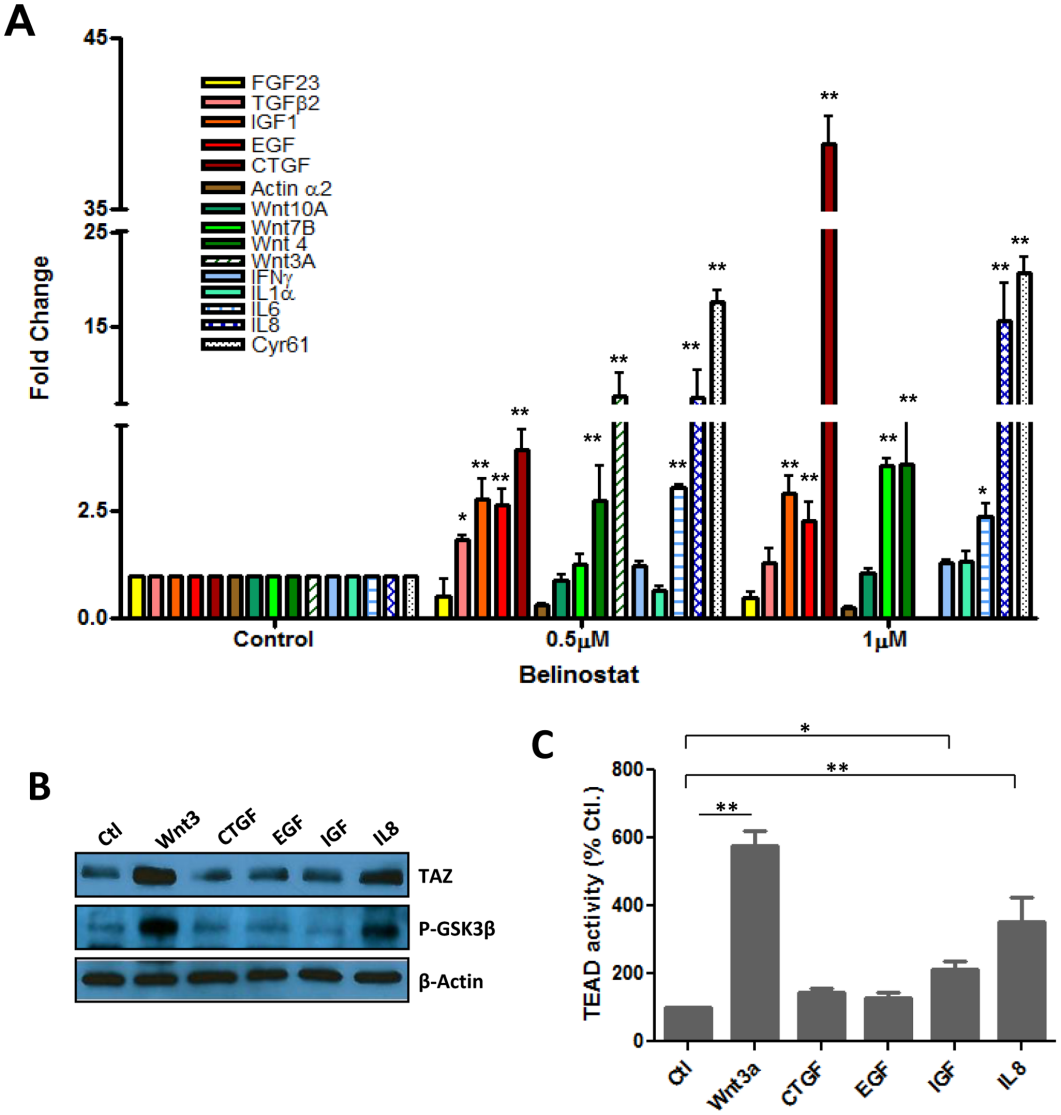
Role of secreted growth factors and cytokines in mediating Belinostat-induced activation of the Hippo pathway. Panel A. SW480 cells were incubated with the indicated concentrations of Belinostat for 24 hours and expression levels of selected secreted factors were determined by QPCR and compared to those in control non-treated cells. Panel B. Effect of individual growth factors and cytokines on TAZ levels and phosphorylation of GSK 3 beta. Cells were incubated with the indicated soluble factors (at 100 ng/ml each) for 24 hours and proteins extracted and processed for western blot using specific antibodies to TAZ and phosphorylated GSK3 beta. Panel C. Effect of individual growth factors and cytokines on activity of the Hippo reporter. Cells transfected with the reporter construct were incubated with the indicated factors for 24 hours and luciferase activity measured as described in the Methods section. Each bar in Panels A and C represents the average of three determinations ±SE. Statistical significance is shown for treated cells compared to the corresponding untreated controls (*p<0.05, **p<0.001).

In light of recent evidence that G protein coupled receptors (GPCRs) may play a key role in mediating the action of soluble factors on the Hippo pathway [36], [37], we determined if inhibition of GPCR signaling by glucagon would reduce the effect of Belinostat on Hippo signaling and TAZ levels. The results presented in Figure 4C indicated that this was not the case and to the contrary glucagon seemed to induce the Hippo reporter activity. This, in addition to the observation that glucagon had no effect on Bel-CM induced TAZ stabilization (Fig. 4D) suggests that the GPCR pathway may not mediate the action of Belinostat on Hippo signaling. Alternatively, the possibility that Belinostat induces expression of growth factors and/or cytokines, which in turn signal for inhibition of the GSK3 beta associated degradation complex and cause stabilization of TAZ seemed plausible (Figure 5A). Data in this figure indicate that exposure of melanoma cells WM 115 to Belinostat resulted in expression of various ligands, some of which are known for their ability to activate Wnt [42], TGF beta [43] or the Hippo signaling pathways [44], [45]. As positive controls, levels of the two downstream genes of Hippo pathway CTGF and Cyr61 increased dramatically (more than 15 times). Others such as Wnt3a and IL8 were induced at levels between 5 to 15 times, while IGF, TGF beta2, Wnt4, Wnt7, Wnt10 and IL6 were induced 2 to 5 times (Fig. 5A). Individual factors were tested for their ability to induce TAZ stabilization and activation of the luciferase reporter and as indicated in Figure 5 B, Wnt3a was the most effective in doing so, consistent with previous reports that the corresponding pathway regulates Hippo signaling [46]. IL8 also enhanced TAZ levels but to a lesser extent, while CTGF, EGF and IGF had no effect in this cellular model perhaps due to lack of expression of the corresponding receptors (Fig. 5B). Interestingly, factors that increased TAZ levels also induced phosphorylation of GSK3 beta, and activity of luciferase (Fig. 5B and 5C) providing further support for the implication of this enzyme in regulation of Hippo signaling.

### Consequences on EMT, Cell Migration and Resistance to Drugs and Potential Role of Targeting GSK3 Beta Associated Degradation Complex in Suppressing these Events

**Figure 6:**
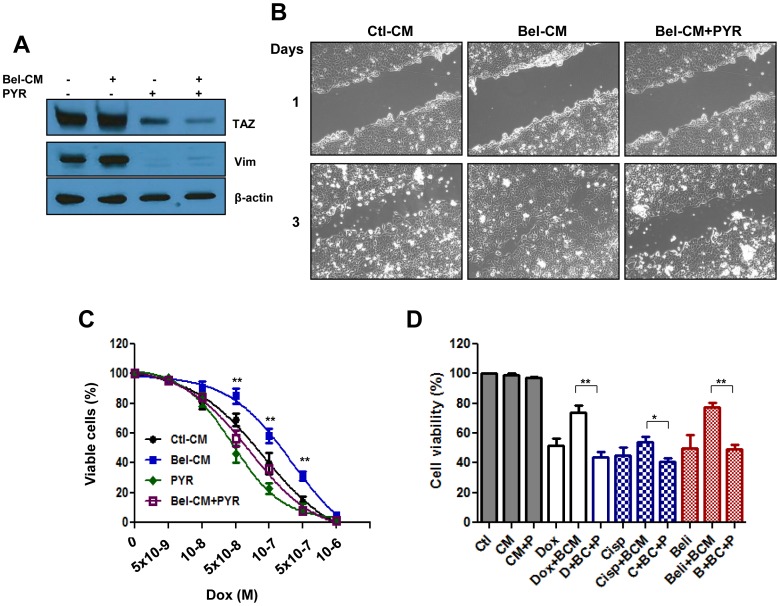
Targeting the GSK 3 beta associated destruction complex reduces TAZ levels, cancer cell migration and resistance to therapy. Panel A. Naïve SW480 cells were exposed to conditioned medium from Belinostat (1 µM) treated counterparts (Bel-CM), in the absence or the presence of Pyrvinium (PYR) at 0.5 µM. After 24 hours, the cells were processed for Western blot with antibodies to TAZ, Vimentin (Vim) and beta actin. Panel B. Monolayer scratch assay depicting the effect of Bel-CM on cell migration and its delay by pyrvinium. MCF cells cultured until confluency and scratches introduces in the monolayer using a pipette tip. The cells were then incubated in the presence or absence of Bel-CM, with or without pyrvinium (0.5 µM) for the indicated times, representative photographs are shown. Panel C. Effect of Bel-CM and pyrvinium of cellular response to doxorubicin. SW480 cells were incubated with doxorubicin at the indicated concentration in absence or presence of Bel-CM, Pyrvinium (PYR) or both. Cell viability was determined by MTT assay as described in the Methods section and the data represented as *per cent* of control non-treated cells. The data represent average of three determinations ±SE. Statistical significance is shown for Bel-CM exposed cells in the absence or the presence of PYR (**p<0.001). Panel D. Effect of PYR on cellular response to other drugs. Cells were exposed to the indicated drugs in the absence or presence of Bel-CM (BCM) and pyrvinium (P). Cell viability was determined by MTT assay after 72 hours in culture. The data represent average of three determinations ±SE. Statistical significance is shown for Bel-CM exposed cells in the absence or the presence of PYR for each drug tested (*p<0.05, **p<0.001).

Tumor microenvironment is known to regulate cancer cell migration and survival. Since most if not all of the secreted factors described above have been reported to play a role in tumor progression, the possibility exist that CM from cancer cells pre-exposed to Belinostat (and thus enriched in these factors) will induce phenotypic features of aggressive cancers such as enhanced expression of EMT genes, cell migration and resistance to drugs. In addition, the finding that GSK3 beta is implicated in histone acetylation mediated transactivation of TAZ suggests that pharmacological targeting of this enzyme or the associated degradation complex should attenuate these effects. We tested these possibilities first by measuring the effect of pyrvinium, a known activator of the GSK3 beta associated degradation complex [47], on the ability of Belinostat to stabilize TAZ and induce the EMT gene Vimentin. As shown in Figure 6A, this compound effectively reduced the level of both genes. In another set of experiments, melanoma cells were exposed to CM from non-treated or from Belinostat-treated counterparts (CM or Bel-CM respectively) and analyzed the consequence of this on cell migration and response to drugs. As shown in Figure 6B, cell migration was accelerated upon exposure to Bel-CM however pyrvinium suppressed it. Bel-CM exposed cells also survived better doxorubicin-mediated toxicity (Fig. 6C) suggesting a protective effect of this medium against chemotherapy. This resistance phenotype is valid for other drugs and can be reversed if cancer cells were pre-incubated with pyrvinium (Fig. 6C and 6D), indicating that this type of compound may represent a useful adjuvant to improve the anticancer activity of TAZ inducing chemotherapeutics.

Overall, findings from this study shed light on a previously unrecognized phenomenon by which certain anti-cancer agents, in particular those that regulate histone acetylation, may paradoxically promote tumor progression by inducing expression of secreted factors capable of stimulating the Hippo pathway and thus facilitating cancer cell migration and resistance to therapy. Since GSK3 beta associated degradation complex is implicated in this process, pharmacological activators of this complex may represent useful tools to improve the anticancer activity of TAZ stabilizing stimuli.

## Discussion

Loss of cell polarity is a hallmark of cancer [48] and activation of the Hippo signaling pathway has emerged as a key regulator of this process [49], suggesting that the associated pathway may represent a compelling target for therapy. For this to be achieved however, the molecular mechanisms that regulate Hippo signaling need to be fully understood. Available data indicate that this pathway is regulated by elements of tight-junctions [48] and G-protein coupled receptors [36]. Here we provide evidence for an additional site of regulation and show that signals from the nucleus, in particular those resulting from changes in histone acetylation may also regulate Hippo signaling. An example of such stimuli is the histone deacetylase inhibitor Belinostat currently used in the clinic to treat cancer [50]. Our findings indicate that Belinostat causes the stabilization of the Hippo transducer TAZ which is known for its oncogenic function and ability to induce cancer stem cell characteristics [23], [24].

The observation that among all stressors tested, inhibitors of histone deacetylases had the most pronounced effects on activity of the Hippo reporter (Fig. 1A) and expression of downstream target genes is in line with the plastic nature of chromatin remodeling and the reversibility of EMT, by comparison to DNA damage which generally leads to a stable phenotype. Interestingly, the effect of Belinostat on TAZ/TEAD reporter activity was not caused by enhanced expression and/or activity of upstream Hippo signaling intermediates such as the kinase core complex (Mst/Lats), however, we noted that this drug induced a concentration-dependent decrease in YAP and increase in TAZ levels in the drug-treated cells (Fig. 2). The reduction of YAP is somewhat intriguing nonetheless it represents a desirable outcome since this gene is known to facilitate cancer progression [27], [51]. In contrast, enhanced TAZ levels in response to Belinostat (Fig. 2) is unwanted for the same reason that this gene is also known to be associated with worst prognosis [23], [24]. Previous work from our laboratory and others have shown that increased histone acetylation promotes EMT, cancer metastasis [52], [53] and resistance to therapy [54], [55], but the underlying mechanism(s) was not understood. Here we show that TAZ may represent the principal mediator of these events and since TAZ and not YAP has been shown to confer cancer stem cell phenotype in breast cancer [33], the latter transcription factor may be dispensable for mediating the pro-EMT effects of HDAC inhibition.

Although histone acetylation is known to be associated with increased gene expression, Belinostat had no effect on TAZ mRNA levels (Fig. 3A). The data revealed that this drug acts on the Akt/GSK3 pathway to prevent TAZ degradation, raising the possibility that secretion of soluble factors which signal for activation of Akt and subsequent inhibition of GSK3 may account for Belinostat-mediated stabilization of TAZ. In support of this, we show (Fig. 4A and 4B) that conditioned medium from cells pre-treated with Belinostat activated the TAZ/TEAD reporter and promoted TAZ stabilization. Apparently, the GPCR pathways does not play a significant role in mediating the effect of Belinostat on the Hippo pathway since cellular treatment with glucagon, a GPCR antagonist, had no effect on TAZ levels or activity of the corresponding reporter (Fig. 4C). We found however that Belinostat-induced expression of several secreted growth factors (Fig. 5A), some of which (i.e. Wnt 3a and IL8) are capable of inducing activity of the Hippo reporter, phosphorylation-mediated inhibition of GSK3 beta and stabilization of TAZ (Fig. 5B and 5C). These findings are in agreement with previous work that both Wnt3a and IL8 signal through GSK3 beta complex [41], [56] and suggest that by integrating different signaling pathways, this complex may represent a conceivable target to inhibit the pro-tumorigenic function of TAZ stabilizing agents. The data also shed light on an inter-cellular cytoprotective mechanism by which histone deacetylase inhibitors activate the Hippo pathway not only in drug affected cells, but also in those that are not directly accessible to the drug. Such a bystander effect is demonstrated by the ability of conditioned medium (Bel-CM) from drug-treated cells, to induce expression of EMT genes and migration in cells not previously exposed to it (Fig. 6A and 6B).

**Figure 7:**
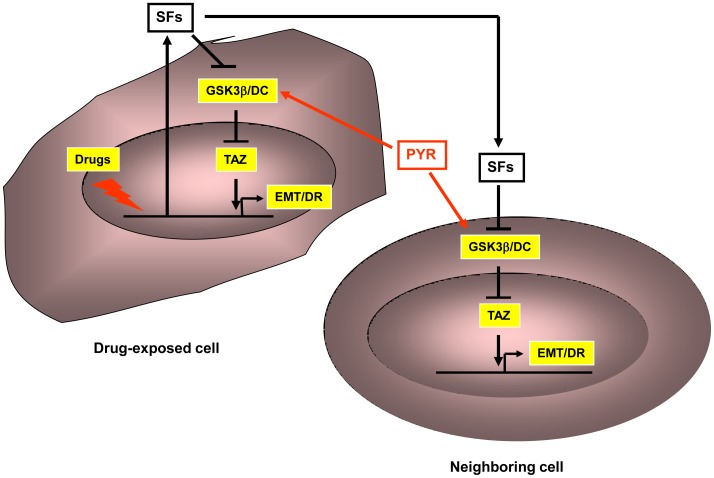
Schematic model depicting nuclear regulation of the Hippo pathway in drug-affected and neighboring cells. Exposure to certain drugs affecting DNA or chromatin results in enhanced expression of secreted growth factors and cytokines. These secreted factors (SFs) may in turn signal in autocrine/paracrine manner for activation of Akt and inhibition of GSK3 beta associated degradation complex (GSK3β/DC) resulting in stabilization of TAZ oncogene. Consequently, expression of EMT genes is enhanced, leading to increased cell migration and drug resistance (DR) in both drug affected and neighboring cells. These processes can be overcome by using pyrvinium (PYR), a pharmacological activator of GSK3 beta associated degradation complex.

Besides metastasis, another cause of cancer recurrence is the development of drug resistance. Over the years, we come to realize that cancer cells have the ability to adapt and ultimately escape the toxicity of virtually any drug tested so far. The underlying mechanisms are numerous and some are well described, however the initial steps leading to development of resistance are not yet understood. In this regard, the finding that increased histone acetylation may induce the development of drug resistance provides a unique opportunity for exploring the causes of this phenomenon in order to prevent its onset. This in addition to the interesting observation that this process is mediated by the GSK3 beta associated degradation complex makes it pharmacologically tractable, for instance by using the recently discovered activator pyrvinium [47] . The data presented in Figure 6 provide proof of concept for this and show that cancer cell exposure to pyrvinium may suppress Belinostat-induced accumulation of TAZ, cell migration and resistance to therapy. Together, findings from this study shed light on a novel mechanism by which certain chemotherapeutic drugs, particularly those that affect chromatin integrity, signal for enhanced migration and survival of both drug affected and neighboring cells (Fig. 7). Since GSK3 beta associated degradation complex was found to play a key role in mediating these events small molecules activators of this complex would represent desirable tools to suppress cancer progression.

## Supporting Information

Data S1 **Quatification of western blot staining from** **Figure 1D** **.** The band intensity was quantified using ImageJ software (NIH). The data represent average of three determinations ±SE. Significance reflects comparison between Belinostat-treated cells and the corresponding non-treated cells (*p<0.05, **p<0.001).
(TIF)
