# Correction: Histone acetylation-mediated regulation of the Hippo pathway

**DOI:** 10.1371/journal.pone.0322605

**Published:** 2025-04-09

**Authors:** Dipanjan Basu, Miguel Reyes-Múgica, Abdelhadi Rebbaa

In Fig 6B of [1], the Day 1 - Bel-CM+PYR panel is incorrect and is a duplicate of the Day 1 - Ctl-CM panel. With this Correction, the authors provide a revised version of Fig 6 from [1] including the correct panel for Day 1 - Bel-CM+PYR. The corresponding author also provided the images underlying the revised Fig 6B in S1 File and stated the original data underlying the rest of the results are unavailable.

**Fig 6:**
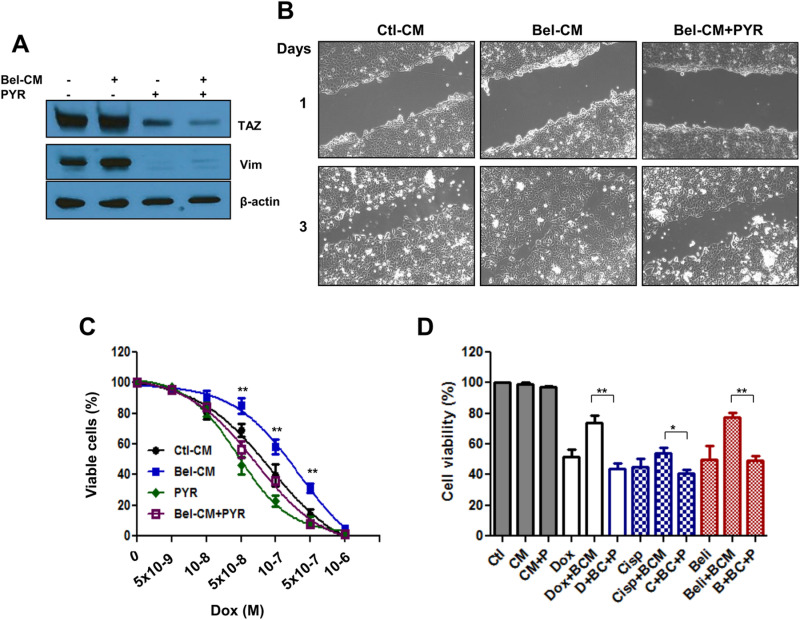
Targeting the GSK 3 beta associated destruction complex reduces TAZ levels, cancer cell migration and resistance to therapy. Panel A. Naïve SW480 cells were exposed to conditioned medium from Belinostat (1 µM) treated counterparts (Bel-CM), in the absence or the presence of Pyrvinium (PYR) at 0.5 µM. After 24 hours, the cells were processed for Western blot with antibodies to TAZ, Vimentin (Vim) and beta actin. Panel B. Monolayer scratch assay depicting the effect of Bel-CM on cell migration and its delay by pyrvinium. MCF cells cultured until confluency and scratches introduces in the monolayer using a pipette tip. The cells were then incubated in the presence or absence of Bel-CM, with or without pyrvinium (0.5 µM) for the indicated times, representative photographs are shown. Panel C. Effect of Bel-CM and pyrvinium of cellular response to doxorubicin. SW480 cells were incubated with doxorubicin at the indicated concentration in absence or presence of Bel-CM, Pyrvinium (PYR) or both. Cell viability was determined by MTT assay as described in the Methods section and the data represented as *per cent* of control non-treated cells. The data represent average of three determinations ±SE. Statistical significance is shown for Bel-CM exposed cells in the absence or the presence of PYR (**p<0.001). Panel D. Effect of PYR on cellular response to other drugs. Cells were exposed to the indicated drugs in the absence or presence of Bel-CM (BCM) and pyrvinium (P). Cell viability was determined by MTT assay after 72 hours in culture. The data represent average of three determinations ±SE. Statistical significance is shown for Bel-CM exposed cells in the absence or the presence of PYR for each drug tested (*p<0.05, **p<0.001).

The authors apologize for the error in the published article.

## Supporting information

S1 File Original images underlying the revised Fig 6B. This file includes the original uncropped images underlying revised Fig 6B of [1]; Ctl-CM Day 1; Bel-CM Day 1; Bel-CM+PYR Day 1; Ctl-CM Day 3; Bel-CM Day 3; Bel-CM+PYR Day 3.
(ZIP)
