# Supplementary figures and images for: Histone Acetylation-Mediated Regulation of the Hippo Pathway
Source: PLoS One. 2013 May 6;8(5):e62478. doi: 10.1371/journal.pone.0062478 (PMC3646011; doi:10.1371/journal.pone.0062478)

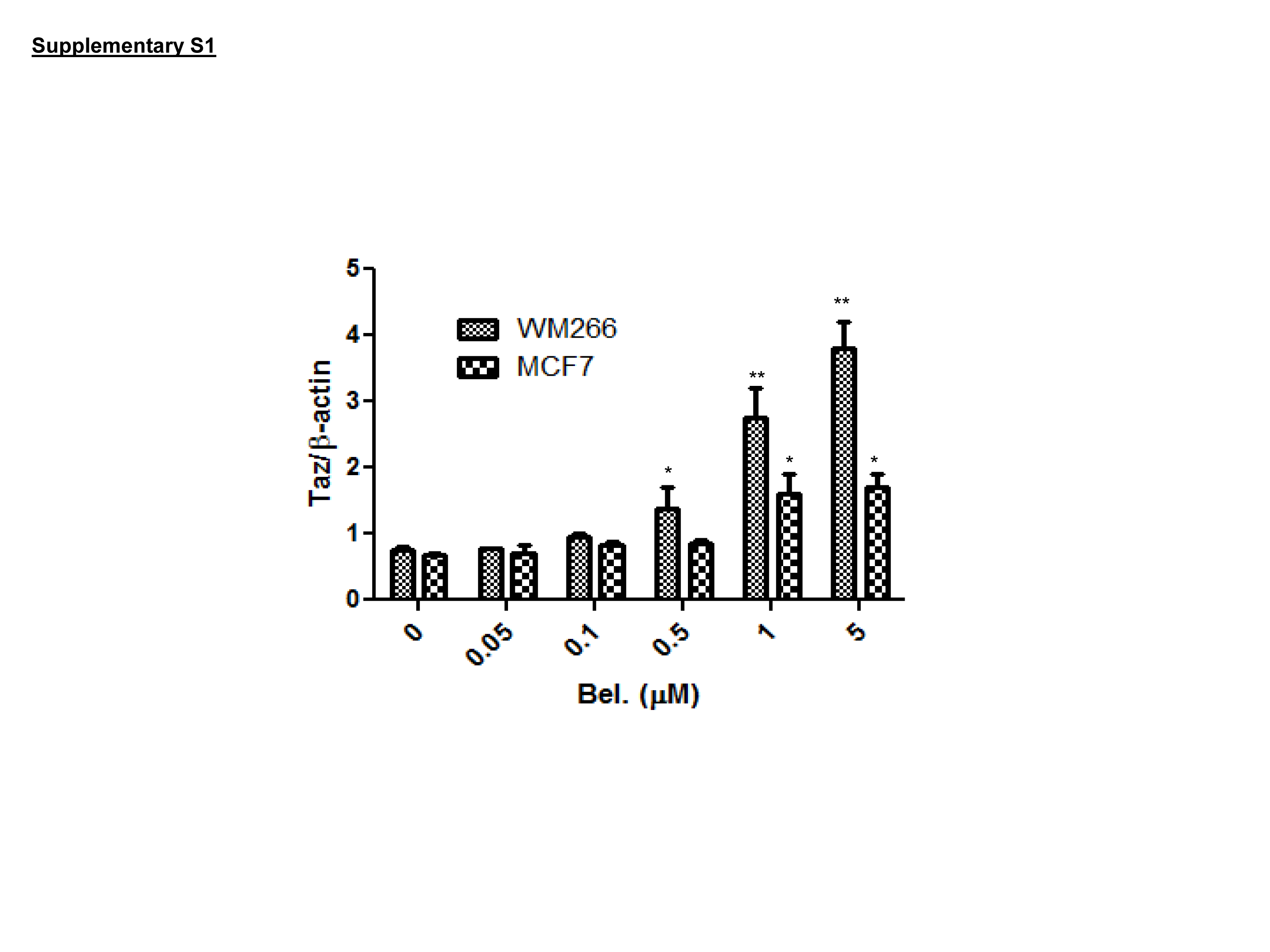

Supplement: Data S1 — Quatification of western blot staining from Figure 1D . The band intensity was quantified using ImageJ software (NIH). The data represent average of three determinations ±SE. Significance reflects comparison between Belinostat-treated cells and the corresponding non-treated cells (*p<0.05, **p<0.001). (TIF) [file pone.0062478.s001.tif]
