# Supplementary figures and images for: Correction: Histone acetylation-mediated regulation of the Hippo pathway
Source: PLoS One. 2025 Apr 9;20(4):e0322605. doi: 10.1371/journal.pone.0322605 (PMC11981133; doi:10.1371/journal.pone.0322605)

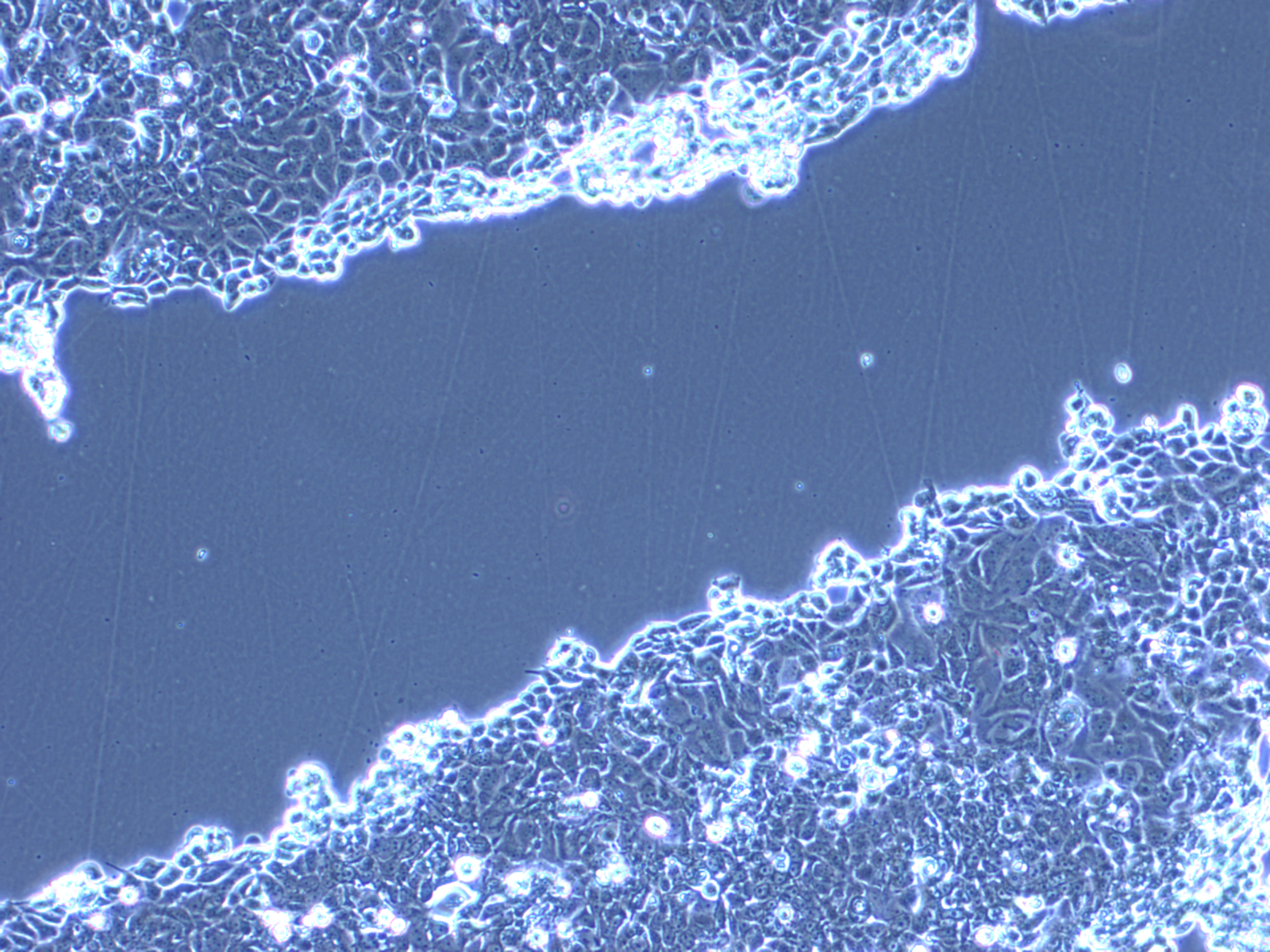

Supplement: S1 File — This file includes the original uncropped images underlying revised Fig 6B of [1]; Ctl-CM Day 1; Bel-CM Day 1; Bel-CM+PYR Day 1; Ctl-CM Day 3; Bel-CM Day 3; Bel-CM+PYR Day 3. (ZIP) [file pone.0322605.s001.zip › S1 File/Bel-CM Day 1.tif]

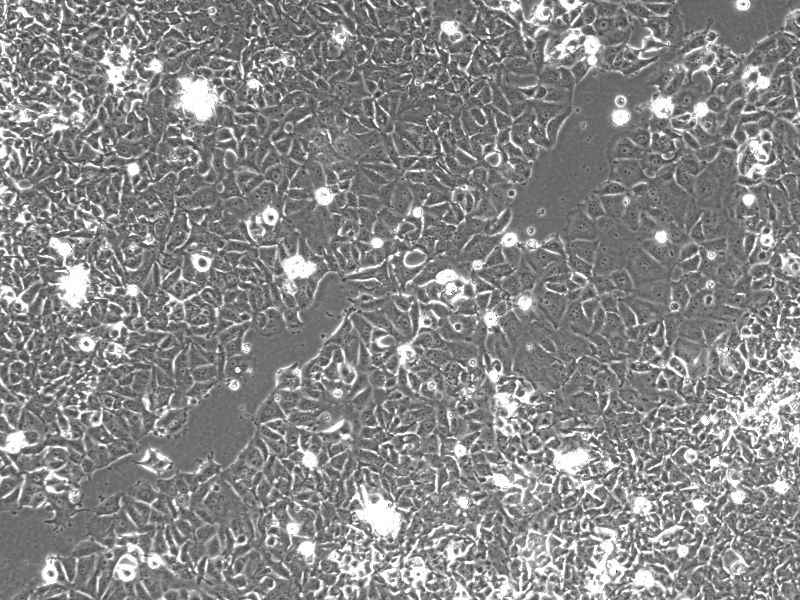

Supplement: S1 File — This file includes the original uncropped images underlying revised Fig 6B of [1]; Ctl-CM Day 1; Bel-CM Day 1; Bel-CM+PYR Day 1; Ctl-CM Day 3; Bel-CM Day 3; Bel-CM+PYR Day 3. (ZIP) [file pone.0322605.s001.zip › S1 File/Bel-CM Day 3.tif]

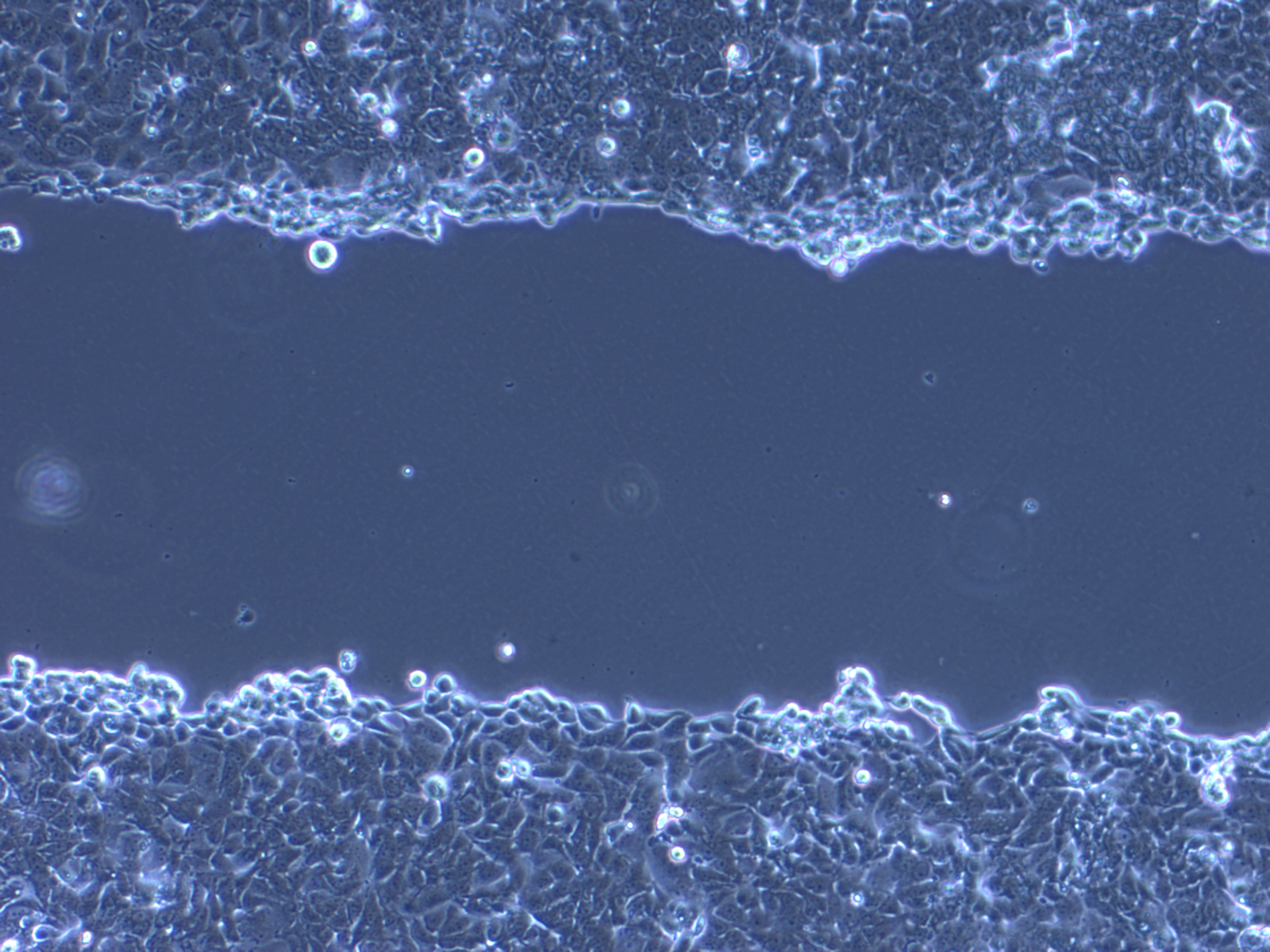

Supplement: S1 File — This file includes the original uncropped images underlying revised Fig 6B of [1]; Ctl-CM Day 1; Bel-CM Day 1; Bel-CM+PYR Day 1; Ctl-CM Day 3; Bel-CM Day 3; Bel-CM+PYR Day 3. (ZIP) [file pone.0322605.s001.zip › S1 File/Bel-CM+PYR Day 1.tif]

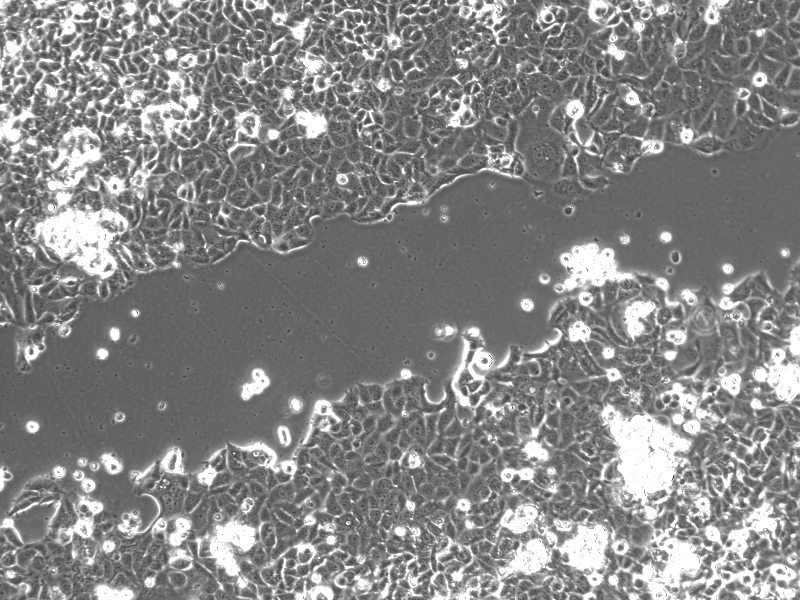

Supplement: S1 File — This file includes the original uncropped images underlying revised Fig 6B of [1]; Ctl-CM Day 1; Bel-CM Day 1; Bel-CM+PYR Day 1; Ctl-CM Day 3; Bel-CM Day 3; Bel-CM+PYR Day 3. (ZIP) [file pone.0322605.s001.zip › S1 File/Bel-CM+PYR Day 3.tif]

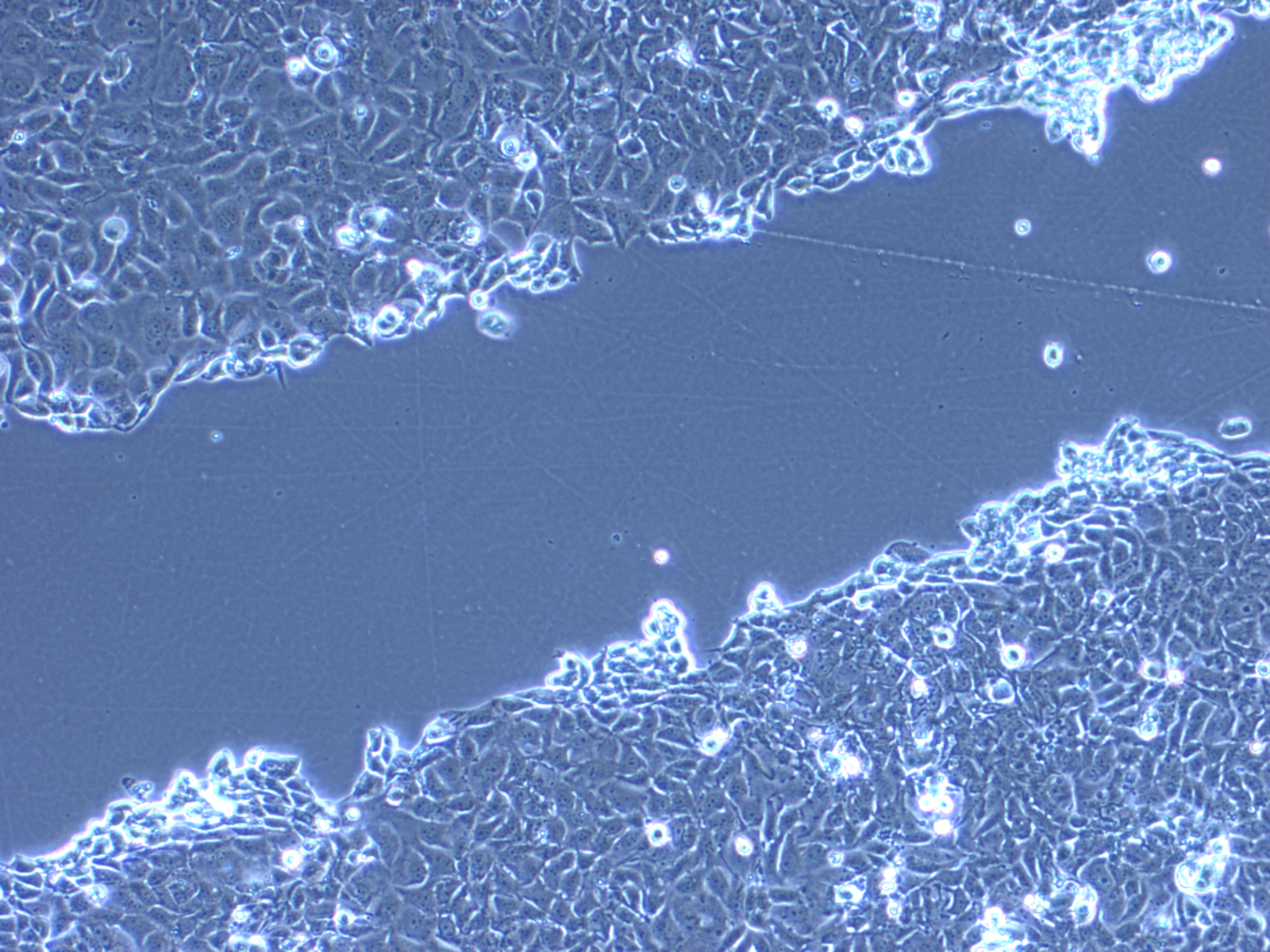

Supplement: S1 File — This file includes the original uncropped images underlying revised Fig 6B of [1]; Ctl-CM Day 1; Bel-CM Day 1; Bel-CM+PYR Day 1; Ctl-CM Day 3; Bel-CM Day 3; Bel-CM+PYR Day 3. (ZIP) [file pone.0322605.s001.zip › S1 File/Ctl-CM Day 1.tif]

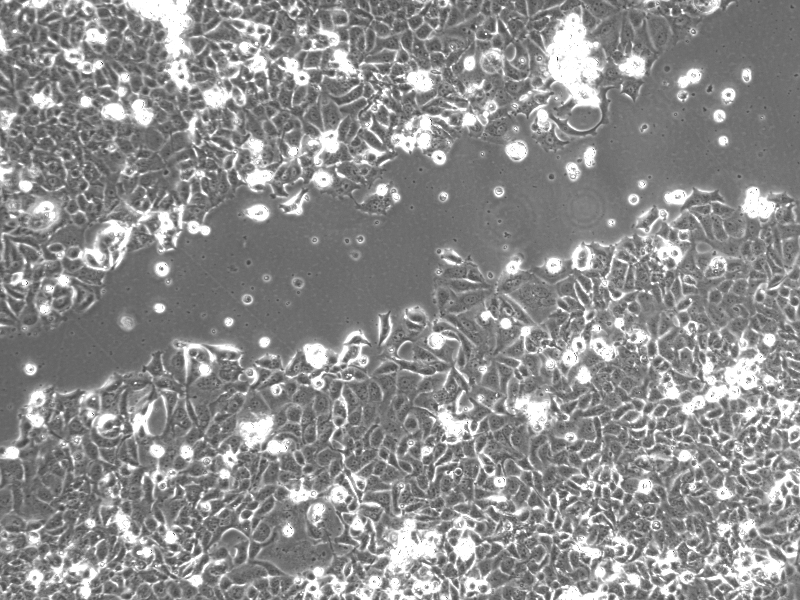

Supplement: S1 File — This file includes the original uncropped images underlying revised Fig 6B of [1]; Ctl-CM Day 1; Bel-CM Day 1; Bel-CM+PYR Day 1; Ctl-CM Day 3; Bel-CM Day 3; Bel-CM+PYR Day 3. (ZIP) [file pone.0322605.s001.zip › S1 File/Ctl-CM Day 3.tif]
